# Associations between vision impairment and multimorbidity among older Chinese adults: results from the China health and retirement longitudinal study

**DOI:** 10.1186/s12877-023-04393-0

**Published:** 2023-10-24

**Authors:** Kun Xiong, Huiyan Mao, Qi’ao Zhang, Changrong Lei, Yuanbo Liang

**Affiliations:** National Clinical Research Center for Ocular Diseases, Eye Hospital, Wenzhou Medical University, No. 270, Xue Yuan Xi Road, Wenzhou, 325027 Zhejiang China

**Keywords:** Vision impairment, Multimorbidity, Chronic conditions, Low- and middle-income countries, Elderly

## Abstract

**Background:**

Although several studies have reported the relationship between vision impairment (VI) and multimorbidity in high-income countries, this relationship has not been reported in low- and middle-income countries. This study aimed to explore the relationship between VI with multimorbidity and chronic conditions among the elderly Chinese population.

**Methods:**

The cross-sectional analysis was applied to data from the China Health and Retirement Longitudinal Study (CHARLS) in 2018. A total of 8,108 participants ≥ 60 years old were included, and 15 chronic conditions were used in this study. Logistic regression analysis was used to analyze the relationship between VI with multimorbidity and chronic conditions.

**Results:**

The prevalence of 15 chronic conditions and multimorbidity was higher among the elderly with VI than those without VI. After adjusting for demographic and socioeconomic confounders, 10 chronic conditions were associated with VI (all *P* < 0.05). Furthermore, positive association was observed between VI and one (odds ratio [OR]: 1.52; 95% confidence intervals [95%CI]: 1.16–2.00; *P* = 0.002), two (OR: 2.09; 95%CI: 1.61–2.71; *P* < 0.001), three (OR: 2.87; 95%CI: 2.22–3.72; *P* < 0.001), four (OR: 3.60; 95%CI: 2.77–4.69; *P* < 0.001), and five or more (OR: 5.53; 95%CI: 4.32–7.09; *P* < 0.001) chronic conditions, and the association increased as the number of chronic conditions (*P* for trend < 0.001). Sensitivity analysis stratified by gender, education, smoking status, and annual per capita household expenditure still found VI to be positively associated with multimorbidity.

**Conclusions:**

For patients older than 60 years, VI was independently associated with multimorbidity and various chronic conditions. This result has important implications for healthcare resource plans and clinical practice, for example, increased diabetes and kidney function screening for patients with VI.

**Supplementary Information:**

The online version contains supplementary material available at 10.1186/s12877-023-04393-0.

## Background

Multimorbidity, defined as the presence of two or more chronic conditions affecting the same individual [1], has become a public health disaster worldwide. It was estimated that more than half of the worldwide population older than 60 years has multimorbidity [2]. Chronic conditions, especially multimorbidity, are associated with decreased quality of life, impaired functional status, poor physical and mental health, and increased mortality [3–7]. As the population rapidly ages, multimorbidity’s public health impact will increase, especially in low- and middle-income countries.

Vision impairment (VI) is also a common condition correlated with aging, and it is among the most disabling chronic conditions for the elderly population. In 2020, about 1.1 billion people suffered from VI worldwide [8], and this number is expected to rise to 1.7 billion by 2050 [8]. Studies have reported that VI and chronic conditions often co-occur [9–11]. Additionally, when combined with VI, chronic conditions significantly affect daily functioning and social participation, while VI may aggravate chronic conditions [12, 13]. Therefore, clarifying the association between VI with multimorbidity and chronic conditions is critical. Such an understanding would contribute to the coordinated healthcare resources plan, thus improving medical practice and care services.

However, most previous studies have focused on the relationship between VI and a single chronic disease. For example, studies have found associations between VI and diabetes, heart disease, cognitive decline, and stroke [14–17]. Few studies have reported on the association between VI with comprehensive chronic conditions and multimorbidity [18–20], and these studies have been conducted in high-income countries. Up to date, no studies have reported on the relationship between VI and multimorbidity in low- and middle-income countries, which may be limited relevance by differences in prevalence and progression rates, the risk factors for VI and chronic conditions, and cultural, lifestyle, environmental, and socioeconomic factors. This relationship is important in low- and middle-income countries, which bear 80% of the global burden of chronic conditions [21], and limited healthcare resources.

Accordingly, the current study aimed to determine the association between VI with multimorbidity and 15 chronic conditions among the elderly Chinese population (≥ 60 years old), using nationally representative survey data pertaining to the world’s largest elderly population of 264 million [22].

## Method

### Study design and population

This cross-sectional study was conducted using data from the China Health and Retirement Longitudinal Study (CHARLS). The CHARLS protocol and population have been described in detail previously [23]. In brief, CHARLS collects high-quality data through face-to-face interviews and structured questionnaires from a nationally representative sample of Chinese residents aged 45 years and older, and its sample is selected using multistage, stratified, proportional-to-scale probability. CHARLS conducted a baseline survey in 2011, which included 17,708 respondents from 450 villages or resident communities in 28 of the 31 provincial-level administrative divisions in mainland China. CHARLS followed up with these participants every two years, and three follow-ups were conducted in 2013, 2015, and 2018, respectively. The CHARLS program’s protocol followed the Declaration of Helsinki and was approved by the Biomedical Ethics Review Committee of Peking University (IRB00001052-11015). All participants signed an informed consent form before completing the survey.

**Fig. 1:**
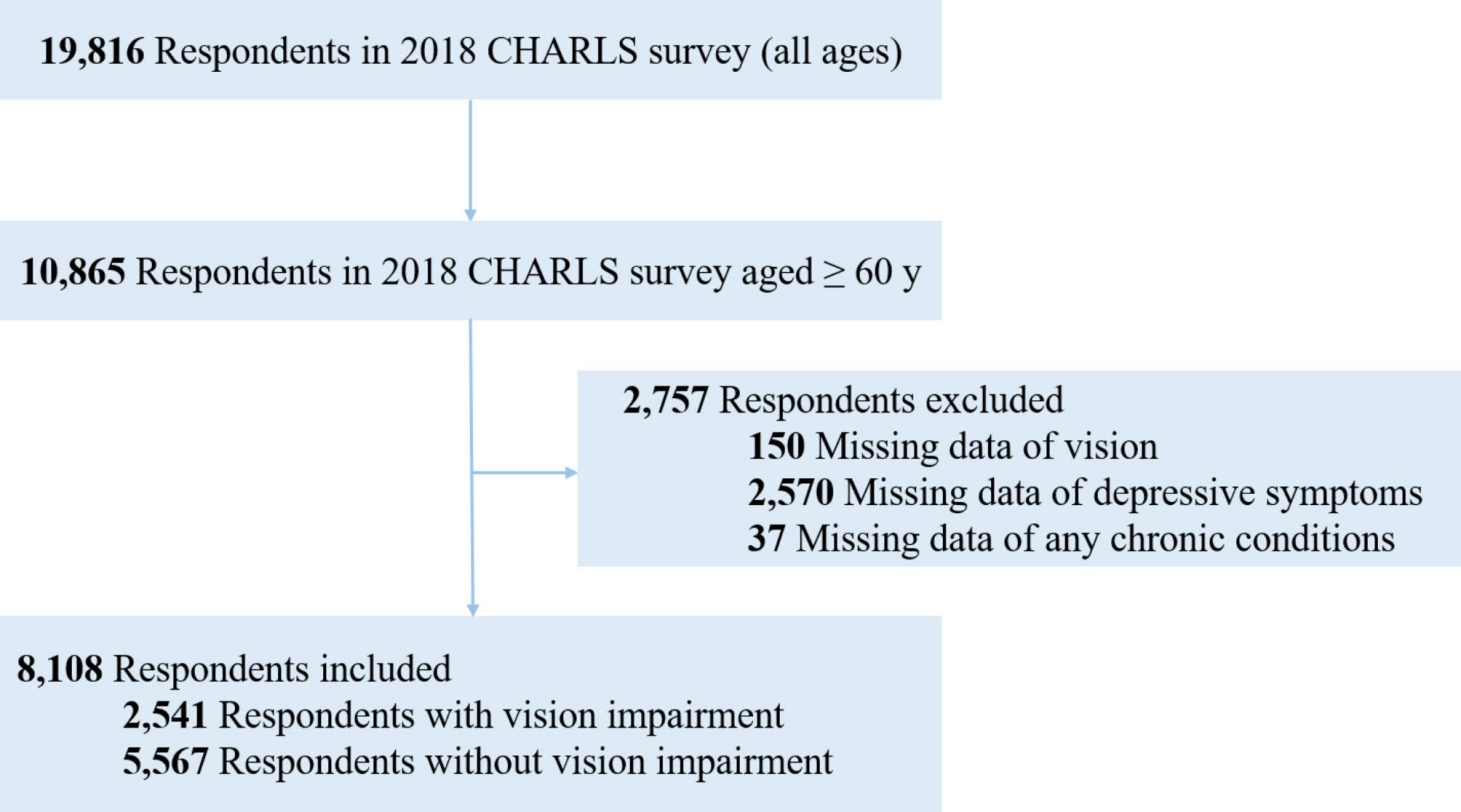
Flowchart depicting this study’s participant selection

In the current study, we used these 2018 data (which included 19,816 participants) to analyze the association between VI and multimorbidity. To focus on age-related VI, we included participants who were aged 60 years and older in our analysis. Participants with missing data on vision, depressive symptoms, or any of the chronic conditions were excluded from this study. Figure 1 is a flowchart depicting the study’s selected participants. The final sample included 8,108 participants with and without VI.

### Defining vision impairment

A self-report assessment of visual function identified VI in the CHARLS data. Participants were asked two questions related to vision impairment: (1) How good is your eyesight for seeing things at a distance, like recognizing a friend from across the street (with glasses or corrective lenses if you wear them)? (2) How good is your eyesight for seeing things up close, like reading ordinary newspaper print (with glasses or corrective lenses if you wear them)? Responses to these questions were “poor,” “fair,” “good,” “very good,” and “excellent”. Participants whose responses were “poor” eyesight for seeing things at a distance or up close were classified as VI in the current study.

### Defining chronic conditions and multimorbidity

We used 15 chronic conditions to measure multimorbidity in this study. Each participant was asked to report whether they had been diagnosed with any of the following chronic conditions by a physician: hypertension, diabetes, dyslipidemia, heart disease, stroke, cancer, chronic lung disease, digestive system diseases, liver disease, kidney disease, asthma, arthritis, and memory-related diseases. The standardized questionnaire assessed chronic conditions in Table S1. Hearing loss was defined as a patient having (a) had hearing problems, (b) worn a hearing aid, or (c) self-reported their hearing status as poor (rather than excellent, very good, good, or fair). Participants with depressive syndrome or emotional, neurological, or mental problems were defined as having psychiatric diseases. Depressive symptoms were assessed by the 10-item Centre for Epidemiologic Studies Depression Scale (CESD-10) was used to assess [24], and participants with the CESD-10 ≥ 10 were defined as having depressive symptoms. Emotional, neurological, or mental problems were from self-reported questions. Multimorbidity was defined as the presence of ≥ 2 chronic conditions in the same person [1].

### Covariates

The CHARLS questionnaire collected data on participants’ demographic and socioeconomic characteristics. These data included age, gender (male or female), residence (rural or urban), marital status (married or partnered versus single or widowed), education levels (primary school or below versus middle school or above), smoking status (current smoker, former smoker, or never having smoked), drinking status (current drinker, former drinker, or never having drunk), and health insurance status (insure or uninsured). Previous studies have confirmed that per capita household consumption expenditure reflects living standards better than household income [25]. Therefore, we used annual per capita household expenditure levels to assess participants’ economic situation. We divided per capita household consumer spending into three groups by tertiles.

### Statistical analysis

The Kolmogorov-Smirnov test was used to verify the continuous variables’ normal distribution. The normally distributed variables were represented using means ± standard deviations (SDs), while the non-normally distributed continuity variables were represented by medians (interquartile ranges). The categorical variables were represented using their number (percentage). An independent-sample *t*-test or chi-square test was used to compare participants’ demographic and socioeconomic characteristics. Two logistic regression models were used to calculate the relationship between VI with multimorbidity and chronic conditions. Model 1 adjusted for age and gender. Meanwhile, Model 2 further adjusted for residence, marital status, education levels, smoking status, drinking status, health insurance, and annual per capita household expenditure levels. To validate the results’ robustness, we performed a sensitivity analysis stratified by gender, education levels, smoking, and per capita household consumption levels. All statistical analyses were performed using Stata (Version 17.0; Stata Corporation, College Station, TX, USA). All *p* values were two-sided, and *P* < 0.05 was considered statistically significant.

## Results

### Participant characteristics

In total, 8,108 participants aged over 60 years enrolled in this study. Participants’ demographic and socioeconomic characteristics are presented in Table 1. Their average age was 68.0 ± 6.1 years, and 48.7% were male. The number of participants with VI stood at 2,541 (31.3%). Generally, compared to the participants without VI, the participants with VI were older and more likely to be female, rural residents, single or widowed, never smoked or drank, and did not have health insurance (all *P* < 0.05). Additionally, the participants with VI had lower education levels and annual per capita household expenditure than those without VI (all *P* < 0.001).

**Table 1:** Characteristics of participants with and without vision impairment

Variable	Total	Vision impairment		
		Yes	No	P-value
No of participants	8,108	2,541	5,567	
Age, y	68.0 ± 6.1	68.5 ± 6.2	67.7 ± 6.0	0.154
Gender				< 0.001
Male	4,161 (51.3)	1,110 (43.7)	3,051 (54.8)	
Female	3,947 (48.7)	1,431 (56.3)	2,516 (45.2)	
Residence				< 0.001
Rural	4,791 (59.1)	1,660 (65.3)	3,131 (56.2)	
Urban	3,317 (40.9)	881 (34.7)	2,436 (43.8)	
Marital status				< 0.001
Married or partner	6,605 (81.5)	2,000 (78.7)	4,605 (82.7)	
Single or widowed	1,503 (18.5)	541 (21.3)	962 (17.3)	
Education level				< 0.001
Primary school or below	5,803 (71.6)	2,004 (78.9)	3,799 (68.2)	
Middle school or above	2,305 (28.4)	537 (21.1)	1,768 (31.8)	
Smoking status				< 0.001
Current	2,257 (27.8)	654 (25.7)	1,603 (28.8)	
Former	1,552 (19.2)	430 (16.9)	1,122 (20.2)	
Never	4,297 (53.0)	1,457 (57.3)	2,840 (51.0)	
Drinking status				< 0.001
Current	2,711 (33.4)	705 (27.7)	2,006 (36.0)	
Former	1,258 (15.5)	434 (17.1)	824 (14.8)	
Never	4,138 (51.0)	1,402 (55.2)	2,736 (49.2)	
health insurance				0.041
Insure	7,829 (96.6)	2,438 (95.9)	5,391 (96.8)	
Uninsured	279 (3.4)	103 (4.1)	176 (3.2)	
Annual household per capita expenditure				< 0.001
Tertile 1	2,271 (33.4)	753 (35.8)	1,518 (32.3)	
Tertile 2	2,270 (33.3)	718 (34.1)	1,552 (33.0)	
Tertile 3	2,270 (33.3)	634 (30.1)	1,636 (34.8)	

Abbreviations: Continuous data are represented as mean (SD); categorical data are represented as the number (percentage)

### Prevalence of multimorbidity and chronic conditions among people with or without VI

**Fig. 2:**
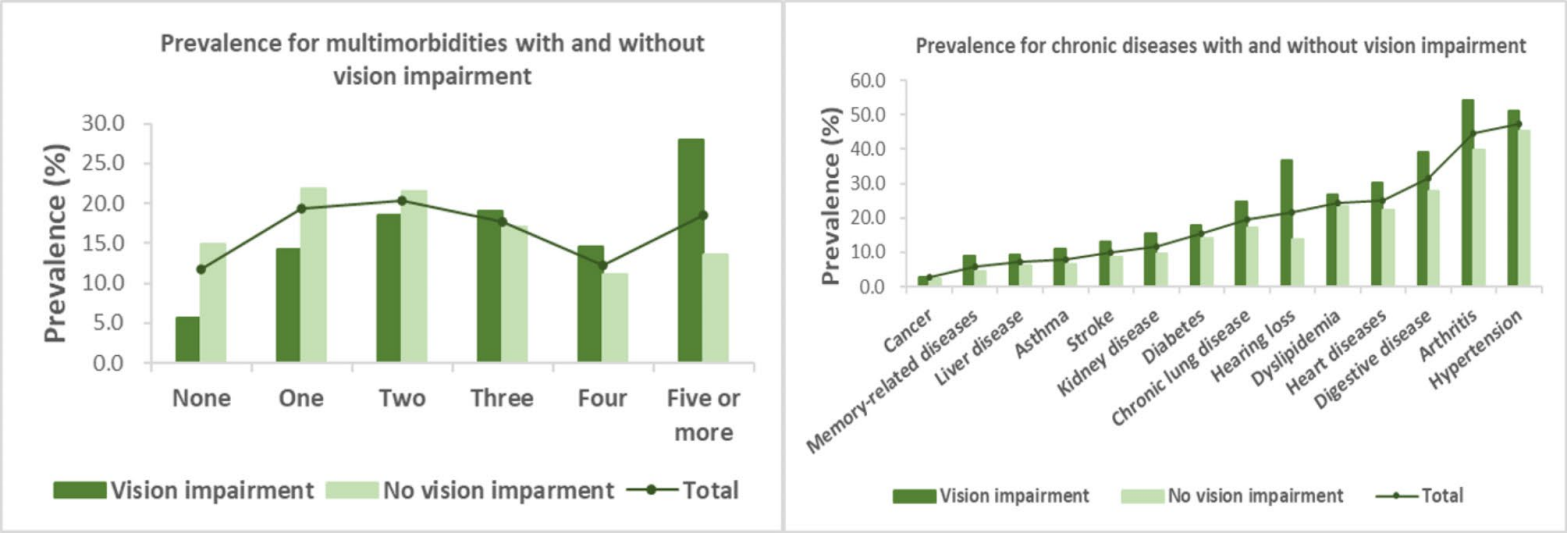
Prevalence for multimorbidities and chronic conditions among people with and without vision impairment

Figure 2 and Table 2 show the prevalence of multimorbidity and chronic conditions among participants with and without VI. Of the participants with VI, 95.8% had at least one chronic condition, compared to 87.5% among the participants without VI (*P* < 0.001). Participants without VI had a higher prevalence of one or two chronic conditions than those with VI (19.6% vs. 11.0% for one condition; 20.9% vs. 15.7% for two conditions). Also, the participants with VI had a higher prevalence of four, or five or more chronic conditions than those without VI (16.2% vs. 12.3% for four conditions; 35.1% vs. 17.6% for five or more conditions). Furthermore, participants with VI had a higher prevalence of the 15 chronic conditions we considered than those without VI, but no statistical difference was observed for cancer (*P* = 0.181). The greatest difference in prevalence between the participants with VI and without VI was observed for hearing loss (31.2% vs. 10.7%) and psychological diseases (52.4% vs. 32.1%).

**Table 2:** Prevalence for multimorbidities and chronic conditions in people with and without vision impairment

	Total	Vision impairment		
		Yes	No	P-value
**Number of chronic conditions**				< 0.001
None	805 (9.9)	107 (4.2)	698 (12.5)	
One	1,372 (16.9)	279 (11.0)	1,093 (19.6)	
Two	1,560 (19.3)	399 (15.7)	1,161 (20.9)	
Three	1,406 (17.3)	452 (17.8)	954 (17.1)	
Four	1,093 (13.5)	411 (16.2)	682 (12.3)	
Five or more	1,872 (22.9)	893 (35.1)	979 (17.6)	
**Chronic conditions**				
Hypertension	3,746 (46.2)	1,261 (49.6)	2,485 (44.6)	< 0.001
Diabetes	1,266 (15.6)	485 (19.1)	781 (14.0)	< 0.001
Dyslipidemia	2,053 (25.3)	715 (28.1)	1,338 (24.0)	< 0.001
Heart diseases	2,041 (25.2)	794 (31.3)	1,247 (22.4)	< 0.001
Stroke	722 (8.9)	297 (11.7)	425 (7.6)	< 0.001
Kidney disease	974 (12.0)	410 (16.1)	564 (10.1)	< 0.001
Liver disease	599 (7.4)	241 (9.5)	358 (6.4)	< 0.001
Chronic lung disease	1,582 (19.5)	631 (24.8)	951 (17.1)	< 0.001
Digestive disease	2,553 (31.5)	994 (39.1)	1,559 (28.0)	< 0.001
Cancer	196 (2.4)	70 (2.8)	126 (2.3)	0.181
Arthritis	3,543 (43.7)	1,363 (53.6)	2,180 (39.2)	< 0.001
Asthma	625 (7.7)	286 (11.3)	339 (6.1)	< 0.001
Hearing loss	1,387 (17.1)	793 (31.2)	594 (10.7)	< 0.001
Memory-related diseases	376 (4.6)	189 (7.4)	187 (3.4)	< 0.001
Psychological diseases	3,124 (38.5)	1,335 (52.5)	1,789 (32.1)	0.001

Abbreviations: Data are represented as the number (percentage)

### Association between VI with Multimorbidity and chronic conditions

Table 3 shows the association between VI with multimorbidity and chronic conditions. After adjusting for age and gender, VI was found to positively correlate with multimorbidities (all *P* < 0.001) and chronic conditions (all *P* < 0.05) except for cancer (*P* = 0.239). After further adjusting for the other potential confounders, this result showed that patients with VI were significantly positively associated with one (odds ratio [OR]: 1.52; 95%CI: 1.16–2.00; *P* < 0.001), two (OR: 2.09; 95%CI: 1.61–2.71; *P* < 0.001), three (OR: 2.87; 95%CI: (2.22–3.72; *P* < 0.001), four (OR: 3.60; 95%CI: 2.77–4.69; *P* < 0.001), and five or more (OR: 5.53; 95%CI: 4.32–7.09; *P* < 0.001) chronic conditions, and this association increased with the number of chronic conditions (*P* for trend < 0.001). Additionally, VI was independently associated with 10 chronic conditions: diabetes, heart disease, stroke, kidney disease, digestive disease, arthritis, asthma, hearing loss, memory-related disease, and psychological diseases (all *P* < 0.05). Among these conditions, hearing loss (OR: 3.19; 95%CI: 2.78–3.67; *P* < 0.001) and psychological diseases (OR: 1.65; 95%CI: 1.47–1.85; *P* < 0.001) were the most strongly associated with VI.

**Table 3:** Association between vision impairment with multimorbidities and chronic conditions

Number of chronic conditions	Model 1 *		Model 2 †	
	OR (95% CI)	P-value	OR (95% CI)	P-value
None	Reference		Reference	
One	1.63 (1.28–2.08)	< 0.001	1.52 (1.16–2.00)	0.002
Two	2.14 (1.69–2.70)	< 0.001	2.09 (1.61–2.71)	< 0.001
Three	2.92 (2.31–3.69)	< 0.001	2.87 (2.22–3.72)	< 0.001
Four	3.71 (2.92–4.71)	< 0.001	3.60 (2.77–4.69)	< 0.001
Five or more	5.43 (4.34–6.79)	< 0.001	5.53 (4.32–7.09)	< 0.001
**Chronic conditions**				
Hypertension	1.17 (1.06–1.28)	0.002	1.01 (0.90–1.14)	0.848
Diabetes	1.39 (1.23–1.58)	< 0.001	1.35 (1.16–1.57)	< 0.001
Dyslipidemia	1.20 (1.08–1.34)	0.001	0.96 (0.83–1.10)	0.545
Heart diseases	1.46 (1.32–1.63)	< 0.001	1.17 (1.02–1.34)	0.021
Stroke	1.60 (1.36–1.87)	< 0.001	1.23 (1.02–1.49)	0.034
Kidney disease	1.74 (1.52–2.00)	< 0.001	1.21 (1.02–1.43)	0.027
Liver disease	1.58 (1.33–1.87)	< 0.001	1.14 (0.93–1.40)	0.219
Chronic lung disease	1.63 (1.45–1.83)	< 0.001	1.10 (0.95–1.28)	0.197
Digestive disease	1.62 (1.46–1.79)	< 0.001	1.29 (1.14–1.46)	< 0.001
Cancer	1.20 (0.89–1.61)	0.239	0.91 (0.63–1.30)	0.588
Arthritis	1.70 (1.54–1.87)	< 0.001	1.26 (1.12–1.42)	< 0.001
Asthma	1.96 (1.66–2.32)	< 0.001	1.43 (1.15–1.78)	0.001
Hearing loss	3.76 (3.33–4.25)	< 0.001	3.19 (2.78–3.67)	< 0.001
Memory-related diseases	2.23 (1.80–2.75)	< 0.001	1.56 (1.21–2.01)	0.001
Psychological diseases	2.23 (2.02–2.46)	< 0.001	1.65 (1.47–1.85)	< 0.001

Abbreviations: OR = odds ratio; 95% CI = 95% confidence interval

*Model 1 adjusted for age and gender

†Model 2 further adjusted for residence, marital status, education level, smoking status, drinking status, health insurance, and annual per-capita household expenditure level

### Sensitivity analysis

To consider the potential implications of participants’ demographic and socioeconomic characteristics, we performed sensitivity analyses stratified by gender, education level, smoking status, and annual per capita household expenditure. The results showed that VI remained significantly associated with multimorbidity (Tables 4 and 5).

**Table 4:** Association between vision impairment and multimorbidities stratified by gender or education level

Number of chronic conditions	OR (95% CI)	P-value	OR (95% CI)	P-value
**Gender** *	**Male**		**Female**	
None	Reference		Reference	
One	1.36 (0.95–1.95)	0.092	1.76 (1.16–2.66)	0.008
Two	1.92 (1.36–2.72)	< 0.001	2.35 (1.58–3.49)	< 0.001
Three	2.78 (1.98–3.91)	< 0.001	3.07 (2.07–4.56)	< 0.001
Four	2.90 (2.03–4.14)	< 0.001	4.54 (3.05–6.78)	< 0.001
Five or more	5.20 (3.73–7.22)	< 0.001	6.13 (4.19–8.96)	< 0.001
**Education level** †	**Primary school or below**		**Middle school or above**	
None	Reference		Reference	
One	1.45 (1.06–1.97)	0.021	1.79 (1.03–3.09)	0.038
Two	2.16 (1.61–2.91)	< 0.001	1.90 (1.11–3.25)	0.019
Three	2.78 (2.06–3.74)	< 0.001	3.23 (1.91–5.44)	< 0.001
Four	3.72 (2.75–5.02)	< 0.001	3.22 (1.85–5.60)	< 0.001
Five or more	5.52 (4.16–7.34)	< 0.001	5.58 (3.36–9.29)	< 0.001

Abbreviations: OR = odds ratio; 95% CI = 95% confidence interval

* Adjusted for age, residence, marital status, education level, smoking status, drinking status, health insurance, and annual per capita household expenditure level

† Adjusted for age, gender, residence, marital status, smoking status, drinking status, health insurance, and annual per capita household expenditure level

**Table 5:** Association between vision impairment and multimorbidities stratified by smoking status or annual per capita household expenditure level

Number of chronic conditions	OR (95% CI)	P-value	OR (95% CI)	P-value	OR (95% CI)	P-value
**Smoking status** *	**Never**		**Former**		**Current**	
None	Reference		Reference		Reference	
One	1.62 (1.10–2.40)	0.016	1.51 (0.75–3.06)	0.252	1.41 (0.91–2.20)	0.124
Two	2.31 (1.59–3.34)	< 0.001	1.85 (0.94–3.63)	0.076	2.01 (1.30–3.10)	0.002
Three	3.25 (2.24–4.71)	< 0.001	2.64 (1.37–5.08)	0.004	2.69 (1.74–4.16)	< 0.001
Four	4.74 (3.25–6.91)	< 0.001	2.84 (1.45–5.54)	0.002	2.74 (1.73–4.33)	< 0.001
Five or more	6.19 (4.33–8.85)	< 0.001	5.01 (2.68–9.35)	< 0.001	5.71 (3.72–8.76)	< 0.001
**Annual per capita household expenditure level** †	**Tertile 1**		**Tertile 2**		**Tertile 3**	
None	Reference		Reference		Reference	
One	1.57 (1.01–2.43)	0.044	1.48 (0.93–2.34)	0.096	1.56 (0.92–2.64)	0.096
Two	2.27 (1.48–3.48)	< 0.001	1.98 (1.28–3.07)	0.002	2.03 (1.24–3.34)	0.005
Three	3.13 (2.04–4.80)	< 0.001	2.53 (1.64–3.91)	< 0.001	2.98 (1.83–4.85)	< 0.001
Four	3.74 (2.42–5.77)	< 0.001	3.34 (2.13–5.23)	< 0.001	3.76 (2.29–6.19)	< 0.001
Five or more	5.91 (3.91–8.96)	< 0.001	5.36 (3.54–8.13)	< 0.001	5.38 (3.37–8.60)	< 0.001

Abbreviations: OR = odds ratio; 95% CI = 95% confidence interval

* Adjusted for age, gender, residence, marital status, education level, drinking status, health insurance, and annual per capita household expenditure level

† Adjusted for age, gender, residence, marital status, education level, smoking status, drinking status, and health insurance

## Discussion

Using nationally representative data on the older Chinese population, this study showed that patients with VI experience a higher prevalence of multimorbidity and chronic conditions than those without VI. After adjusting for residence, marital status, education levels, smoking status, drinking status, health insurance, and annual per capita household expenditure levels, we found that VI was independently associated with multimorbidity and various chronic conditions, and this association increased with the number of chronic conditions. Additionally, this association was not influenced by gender, educational levels, smoking status, or annual per capita household expenditure. To the best of our knowledge, this study is the first to explore the relationship between VI with multimorbidity and extensive chronic conditions in low- and middle-income countries.

This study analyzed the association between VI with extensive chronic conditions and multimorbidity, highlighted the cumulative effect of chronic conditions. Court et al. reported a significant association between VI and multimorbidity [18]. However, this study did not fully correct for demographic and socioeconomic confounders. Furthermore, a National Health Interview Survey in the United States found that patients with four patterns of multimorbidity group had a higher risk of VI compared with the healthy group [20]. This current study first analyzed the relationship between VI with multimorbidity in low- and middle-income countries, and we have corrected for potential confounding factors and performed sensitivity analysis. Our study provides robust evidence for the relationship between VI and multimorbidity. In addition, our results showed a stronger relationship between VI and multimorbidity compared with two previous studies in high-income countries [18, 19], which may be related to harder access to healthcare in low-and middle-income countries. However, this relationship’s underlying mechanism remains unclear. The accumulation of multiple deficits and the interaction between multiple domains may be important factors in the high risk of adverse health outcomes [26]. Therefore, individuals with multiple chronic conditions are more likely to develop VI due to the accumulated risk caused by pathological pathways such as vascular damage, neurodegeneration, inflammatory response, and immunity.

This study showed that the association between VI and multimorbidity is stronger among women. An important reason may be gender inequality in health insurance and health care; women have less health insurance and are less likely to receive health care than men [27, 28]. Studies also confirmed that persons with females are more susceptible to VI [29, 30]. In addition, chronic conditions and VI are bidirectional causal associations [31]. The above reasons may prompt this association to be stronger in women. Our result also showed that individuals with lower education levels are more likely to have VI, which may be that they are less likely to have health insurance or healthcare and worse health conditions [32, 33].

Previous studies have reported that VI is associated with specific chronic conditions [14–16, 34]. Our study shows that stroke and VI are associated, consistent with previous clinical studies [17, 35]. Further, the current population study confirms that patients with stroke should conduct visual function assessments. We also found strong associations between arthritis and VI. Arthritis is a common autoimmune disorder associated with sight-threatening ocular diseases such as uveitis, keratitis, and corneal melts [36]. Moreover, patients with arthritis usually use long-term corticosteroid therapy, and studies confirmed that corticosteroids are a risk factor for glaucoma and cataracts [37, 38]. Moreover, VI often co-occurs in elderly patients with hearing loss, and both diseases were considered important barriers to physical activity in low-and middle-income countries [39]. Therefore, good health services are needed for patients with VI and hearing loss to improve their health status and quality of life. At present, mental problems are a great global public health concern, and our study showed a 0.6-fold increased risk of psychological disorders in patients with VI, consistent with previous reports [40, 41], which reminds the whole society and family to pay attention to the mental health of patients with VI.

The causal relationship between VI and chronic conditions is complex. The Lancet Global Health Commission recently presented integrative evidence regarding the relationship between VI and chronic conditions [31]. The commission proposed that some chronic conditions cause VI, including diabetes, hypertension, and hyperlipidemia [42–44]. Additionally, VI and chronic conditions may share common risk factors. For example, drinking alcohol is associated with systemic diseases (such as liver, digestive, and cardiovascular diseases) [45], as well as ocular abnormalities (such as diabetic retinopathy, age-related macular degeneration, and glaucoma) [46]. Importantly, VI and chronic conditions can be linked by mediating factors likely limited physical activity, reduced access to health care, and increased social isolation [31], leading indirectly to or exacerbating chronic conditions.

Mortality due to chronic conditions accounts for three-quarters of all global deaths, with low- and middle-income countries accounting for 77% [47]. Therefore, to cope with the burden of multimorbidity, the healthcare systems should transform from a single-disease model to a more effective management multimorbidity model. Clinical guidelines have developed in the United States and Europe [48, 49], emphasizing patient-centered healthcare for older adults with multimorbidity. This current study showed that 85% of patients with VI Combined with multimorbidity. Previous studies have confirmed that VI, combined with chronic conditions, influences patients’ health status and quality of life significantly more than one condition alone [9, 50]. Additionally, studies have suggested that patients with VI have less access to healthcare than those without VI [51, 52]. Therefore, for patients who have both VI and systemic conditions, especially multimorbidity, clinicians should pay more attention to their healthcare services to improve their physical status. However, the current study did not analyze the healthcare utilization and health expenditure of patients with both VI and multimorbidity or chronic conditions, and these elements require further research.

This study has several strengths. First, it is the first nationally representative study to explore the association between VI with multimorbidity and 15 chronic conditions among the Chinese population. Second, this study adjusted for demographic and socioeconomic confounding factors and explored whether gender, education levels, smoking, and annual per capita household expenditure altered the association between VI and multimorbidity. Nonetheless, this study also faced several limitations. First, as a cross-sectional study determining the association between VI with multimorbidity and chronic conditions, it has not established causes or effects, and longitudinal studies are needed to clarify causality. Second, although previous studies have shown that self-reporting is satisfactory for chronic conditions compared to physician diagnoses [53], self-reporting may have recall and other biases, which might have underestimated chronic conditions’ prevalence. Third, although we corrected potential confounders such as age, gender, education levels, etc., other residual confounders may not be included in our study. In addition, we may have included non-confounding in this analysis. Finally, the CHARLS visual function assessment was conducted using a visual acuity questionnaire rather than objectively measured. Although previous studies have confirmed that self-reported visual acuity is a good indicator of overall visual function and a strong association between self-reported visual acuity and objective measurements [54, 55], some discrepancies may exist.

## Conclusion

In this study, we found a higher prevalence of chronic conditions among elderly Chinese patients with VI than those without VI. Furthermore, VI was independently associated with multimorbidity, and this association was found to increase with the number of chronic conditions. These results indicate that VI often coexists with multiple chronic health conditions in low- and middle-income countries, patients with multimorbidity will benefit from eye care, and this will improve the healthcare system’s efficiency. This has important implications for the healthcare services plan and clinical practice.

### Electronic supplementary material

Below is the link to the electronic supplementary material.

Supplementary Material 1

## Data Availability

All data collected in the CHARLS are maintained at the National School of Development of Peking University, Beijing, China. The datasets are available from https://charls.pku.edu.cn/pages/data/111/zh-cn.html.
