# Supplementary material for: Associations between vision impairment and multimorbidity among older Chinese adults: results from the China health and retirement longitudinal study
Source: BMC Geriatr. 2023 Oct 24;23:688. doi: 10.1186/s12877-023-04393-0 (PMC10594768; doi:10.1186/s12877-023-04393-0)
Supplement: Supplementary file 1 — Supplementary Material 1 [file 12877_2023_4393_MOESM1_ESM.docx]

**Supplementary Table S1.** The questions were used to assess chronic conditions.

| **Questions** | **Answers** |
| --- | --- |
| Have you been diagnosed with [conditions listed below, read one by one] by a doctor?  [IWER: Read one by one. 1=yes, 2=no.] | (1) Hypertension  (2) Dyslipidemia (elevation of low density lipoprotein, triglycerides (TGs), and total cholesterol, or a low high density lipoprotein level)  (3) Diabetes or high blood sugar  (4) Cancer or malignant tumor (excluding minor skin cancers)  (5) Chronic lung diseases, such as chronic bronchitis, emphysema (excluding tumors, or cancer)  (6) Liver disease (except fatty liver, tumors, and cancer)  (7) Heart attack, coronary heart disease, angina, congestive heart failure, or other heart problems  (8) Stroke  (9) Kidney disease (except for tumor or cancer)  (10) Stomach or other digestive disease (except for tumor or cancer)  (11) Emotional, nervous, or psychiatric problems  (12) Memory-related disease  (13) Arthritis or rheumatism  (14) Asthma |
